# Influences of Microscopic Imaging Conditions on Accuracy of Cell Morphology Discrimination Using Convolutional Neural Network of Deep Learning

**DOI:** 10.3390/mi13050760

**Published:** 2022-05-11

**Authors:** Masashi Yamamoto, Shogo Miyata

**Affiliations:** 1 Graduate School of Science and Technology, Keio University, 3-14-1 Hiyoshi, Kohoku-ku, Yokohama 223-8522, Kanagawa, Japan; ironmasashi3434@a3.keio.jp; 2 Faculty of Science and Technology, Keio University, 3-14-1 Hiyoshi, Kohoku-ku, Yokohama 223-8522, Kanagawa, Japan

**Keywords:** image-based discrimination, myoblast, C2C12, optical magnification

## Abstract

Recently, automated cell culture devices have become necessary for cell therapy applications. The maintenance of cell functions is critical for cell expansion. However, there are risks of losing these functions, owing to disturbances in the surrounding environment and culturing procedures. Therefore, there is a need for a non-invasive and highly accurate evaluation method for cell phenotypes. In this study, we focused on an automated discrimination technique using image processing with a deep learning algorithm. This study aimed to clarify the effects of the optical magnification of the microscope and cell size in each image on the discrimination accuracy for cell phenotypes and morphologies. Myoblast cells (C2C12 cell line) were cultured and differentiated into myotubes. Microscopic images of the cultured cells were acquired at magnifications of 40× and 100×. A deep learning architecture was constructed to discriminate between undifferentiated and differentiated cells. The discrimination accuracy exceeded 90% even at a magnification of 40× for well-developed myogenic differentiation. For the cells under immature myogenic differentiation, a high optical magnification of 100× was required to maintain a discrimination accuracy over 90%. The microscopic optical magnification should be adjusted according to the cell differentiation to improve the efficiency of image-based cell discrimination.

## 1. Introduction

For clinical applications in regenerative medicine, it is necessary to proliferate a large number of cells while still maintaining their functions as being suitable for therapy. In the case of pluripotent stem cells (PSCs), such as induced PSCs, it is necessary to proliferate a large number of cells while maintaining their undifferentiated states. Automated cell culture devices have been developed for the expansion of PSCs [1] and are commercially available. PSCs are easily affected by the surrounding environment and culturing procedures; in this context, physical disturbances such as temperature changes and vibrations can cause PSCs to degenerate and fail to maintain their undifferentiated states, resulting in a loss of pluripotency. Therefore, it is important to evaluate the cell quality (e.g., by evaluating cells’ pluripotency). Conventional methods for determining cell quality, such as those based on polymerase chain reactions, enzyme-linked immunosorbent assays, and histological evaluation methods with fluorescence staining, are generally invasive. In general, non-invasive evaluation methods are more desirable for cell quality assessments in automated culture systems.

The most common method for obtaining a non-invasive evaluation of cell functionality is to observe a cell’s morphology in bright field images using an optical microscope. However, it is difficult to assess cell qualities (phenotype, function, etc.) based only on microscopic images without biochemical labeling (such as fluorescent staining), even for a skilled technician. This is because living cells have rheological properties that dynamically change their morphologies according to their phenotypes and functions [2]. Considering this situation, Diane et al. reported the effectiveness of automated discrimination techniques using image processing based on machine learning, thereby avoiding a dependence on the subjectivity or experience of technicians [3]. Among such techniques for image-based discrimination, deep learning-based methods, which generate discrimination metrics by extracting features from large amounts of image data, have attracted considerable attention [4]. In cell discrimination based on image processing with machine learning, the optimization of the architecture and parameters is important for improving the discrimination accuracy [5,6]. In image analysis based on deep learning, it is necessary to acquire a large amount of image data to provide efficient training for extracting the features of the image data. Furthermore, to accurately evaluate cells during mass culturing in automated culture devices, it is necessary to rapidly obtain a number of microscopic images in different culture vessels during the culturing. Therefore, reducing the time required to acquire microscopic images is important for improving the efficiency of cell discrimination by deep learning.

Although there have been studies [7] on improving efficiency when collecting image datasets for machine learning, these studies have mainly focused on data extraction algorithms (such as those based on cell image segmentation), and not on experimental approaches. In addition, no research has focused on improving the efficiency of acquiring cell microscopic image data for machine learning by considering the time and acquisition conditions required for obtaining such microscopic images. In general, the time required to acquire a microscopic image depends on the optical magnification of the microscope. This is because a lower optical magnification of the microscope enables a wider field of view and a corresponding reduction in the image acquisition time. However, a lower optical magnification decreases the resolution of the microscopic image and can therefore lead to the loss of detailed information on the cell morphology within the microscopic image.

In this study, we aimed to elucidate the effects of the imaging conditions in phase contrast microscopy (as commonly used in cell culturing processes) on the accuracy of cell morphology discrimination. As a fundamental study, the C2C12 cell line was used for myoblast differentiation culturing. C2C12 cells are mouse skeletal myoblast cells. They change their phenotypes during the differentiation process from a round shape to an elongated tubular shape. From the acquired image data, a cell differentiation discrimination system was established based on deep learning. As a training dataset for the deep learning, cell microscopic images were obtained at two different optical magnifications, and the effect of the imaging magnification on the discrimination accuracy was investigated.

## 2. Materials and Methods

### 2.1. C2C12 Cell Culture and Myotube Differentiation

In this study, mouse myoblasts (C2C12 cell line; RCB0987, RIKEN Cell Bank, Ibaraki, Japan) were cultured to construct a dataset for training with a deep learning algorithm and evaluating the discrimination accuracy. During myogenic differentiation, C2C12 cells change their morphologies to a spindle shape and form myotube-like structures. As C2C12 cells are a general cell line that is used to observe undifferentiated and myogenic morphologies by phase-contrast microscopy, microscopic images of the C2C12 cells were acquired to construct the image dataset for the deep learning and its verification. 

The C2C12 cells were initially proliferated in Dulbecco’s Modified Eagle Medium (DMEM) high glucose (12100-046, Gibco, Waltham, MA, USA) +10% fetal bovine serum +1% antibiotic-antimycotic (09366-44, Nacalai Tesque, Kyoto, Japan) as a maintenance medium. After proliferation, the C2C12 cells were differentiated in DMEM high-glucose +2% horse serum +1% antibiotic-antimycotic as a myogenic differentiation medium [8]. The cells were cultured in maintenance medium for 2 days to grow to confluence, and then were cultured in the myogenic differentiation medium to initiate myogenic differentiation. The start date of the myogenic differentiation was defined as day 0. The culture medium was changed every 2 days, and 50 phase-contrast microscopy images were taken on days 3, 6, 9, and 12. To evaluate the effect of the optical resolution on the discrimination accuracy of the cell phenotype, the microscopy images were acquired at 40× and 100× optical magnifications, respectively. A 4× objective lens (NA = 0.13; UPLFLN 4×, Olympus, Tokyo, Japan), 10× objective lens (NA = 0.25; CACHN 10× PHP; Olympus, Tokyo, Japan), and 10× eyepiece lens (WH 10×; Olympus, Tokyo, Japan) were used. A phase contrast microscope (CKX41, Olympus, Tokyo, Japan) and charge-coupled device camera (DP72, Olympus, Tokyo, Japan) were used to obtain the images. 

**Figure 1:**
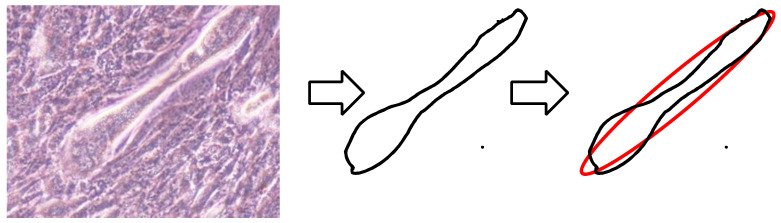
Measurement of major and minor axes of myotube-like cells using ellipse approximation.

The C2C12 cells are elongated by differentiation to form myotube-like morphologies in the myogenic differentiation medium. An ellipse approximation was performed for each cell, and the length of the major axis and aspect ratio were used as the evaluation indices for the geometric properties of the cells. As shown in Figure 1, the outer edges of the cells were extracted on each day, and the ellipse approximation was performed using ImageJ software (V1.52, NIH, Bethesda, MD, USA). The ellipse approximation was performed for 50 cells in each image, and the major axis length and aspect ratio (major axis/minor axis) were calculated. In this study, the length of the long axis was used as an evaluation index for the cell size, and the aspect ratio was used as an index for the cell morphology.

### 2.2. Preparation of Training Data from Cell Microscopic Image Data

The obtained microscopic image data were sufficiently large to increase the computational cost for training with a deep learning algorithm. Therefore, the images were preprocessed before training as follows. First, the lengths of the major and minor axes of the myotube-like cells were measured by approximating them with an ellipse on days 3 and 6, as the boundaries of each cell could be individually recognized. To prepare the training dataset, images for the training dataset were trimmed randomly from original microscopic images to a square of 1.2 times the average length of the long axis, as measured in the procedure described above. This process was performed for images at all optical magnifications and on all days to create a dataset of 600 images. The images on days 9 and 12 were randomly cropped to the same field of view size on day 6, as the ellipse approximation was difficult for fully differentiated myotube-like cells. The cells on day 0 were defined as undifferentiated cells, and those on days 3, 6, 9, and 12 were defined as cells in the process of myogenic differentiation. The microscopic images on day 0 were also cropped to create a training image dataset of undifferentiated cells. By performing the above preprocessing on the microscopic images, a total of 1200 images were obtained for the training dataset (including those from days 3, 6, 9, and 12), with 600 undifferentiated cell images and 600 myotube-like cell images.

### 2.3. Cell Assessment System Using Deep Learning

**Figure 2:**
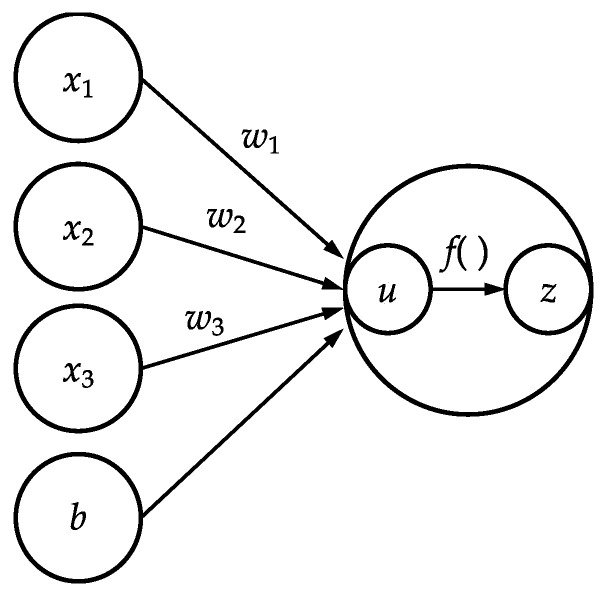
Basic calculation algorithm of deep learning.

For image processing in deep learning, the image is considered as a matrix of pixel values, and discrimination is performed by repeatedly performing the arithmetic operations shown in Equations (1) and (2) (Figure 2).

(1) $$u=w_{1}x_{1}+w_{2}x_{2}+w_{3}x_{3}+b$$

(2) $$z=f\left(u\right)$$

**Figure 3:**
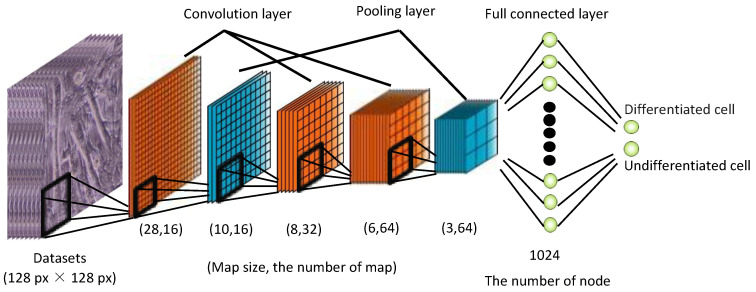
Architecture of the convolutional neural network model used in this study.

In the above, *x* is the luminance value of the pixel; *w* and *b* are the weights and biases, respectively, as optimized by learning; and *f* is the activation function. In this learning model, the cell image was considered as being in an undifferentiated state if the last computed value of *z* was zero, and as being in a differentiated state if it was one. Training with deep learning involved iteratively adjusting *w* and *b* to output the correct answer. The activation function in the deep learning framework works in the same manner as the action potential in neurons. The activation function only outputs when the input exceeds the threshold. The sigmoid and rectified linear unit (ReLU) functions are commonly used as activation functions. In a sigmoid function, the output varies gradually and smoothly with respect to the input. When the absolute value of the input is large, the output saturates to a constant value [9]. In the ReLU function, learning progresses quickly owing to the large difference between the output and the input, and the computational cost for learning is lower [10]. In our learning model for cell image discrimination, the ReLU function was used as the activation function, owing to its fast-learning progress. In deep learning, the most common learning model used for image discrimination is the convolutional neural network (CNN) model, which extracts features by repeatedly applying filters to a component matrix of pixel values [11]. In this study, the learning model was constructed based on AlexNet [12], a CNN model. As shown in Figure 3, the model used in this study comprised three layers for convolution, two layers for pooling, and two layers for providing the total combination. To implement the deep learning model, we used Chainer (from Preferred Networks), an open-source framework for computing and training neural networks. In our learning model, the number of layers was set to being relatively small to prevent overtraining.

### 2.4. Evaluation of Discrimination Accuracy for Cell Differentiation

In this study, the captured images were divided to generate two datasets to evaluate the discrimination accuracy: one for training and the other for validation. The discrimination accuracy was evaluated during the learning process. The set of 1200 cell microscopic images was divided into 900 images for training, 100 images for validation, and 200 images for the final validation of the discrimination accuracy. The images of differentiated and undifferentiated cells were evenly distributed in each dataset. The model exhibiting the best discrimination accuracy in the validation process was adopted as the learning model for the cell image discrimination. Ultimately, 200 images were obtained for the final validation of the discrimination accuracy, and the correct response rate was evaluated as the accuracy of our image-based cell discrimination system.

## 3. Results and Discussion

### 3.1. Extraction of Shape Changes Owing to Myotube Differentiation of Cells

**Figure 4:**
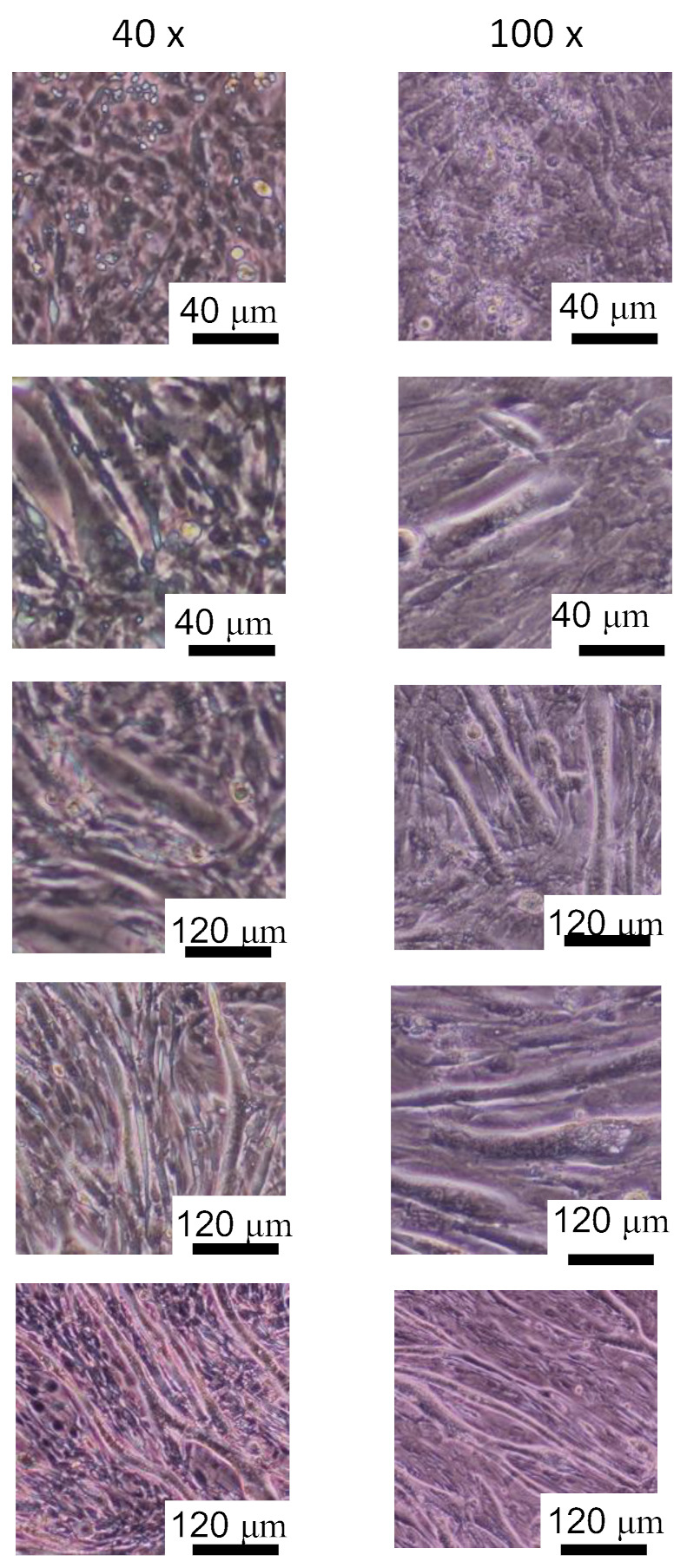
Microscopic images of C2C12 cells during myogenic differentiation culture.

**Figure 5:**
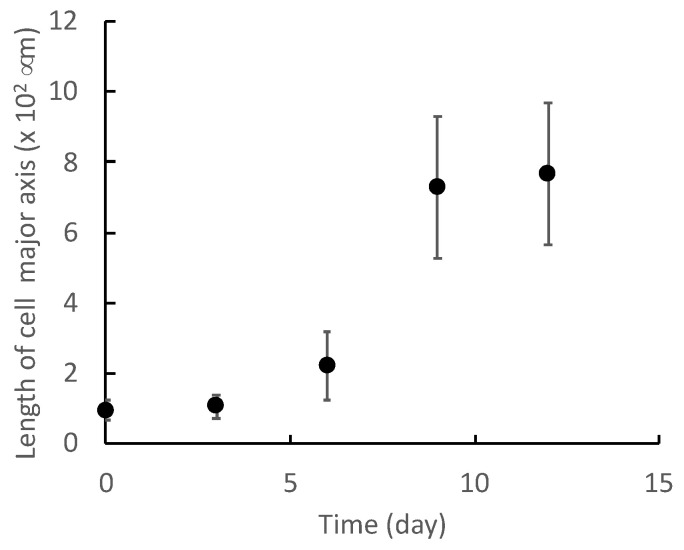
Change in major axis of C2C12 cells during myogenic differentiation culture. Mean +/− SD, n = 25.

**Figure 6:**
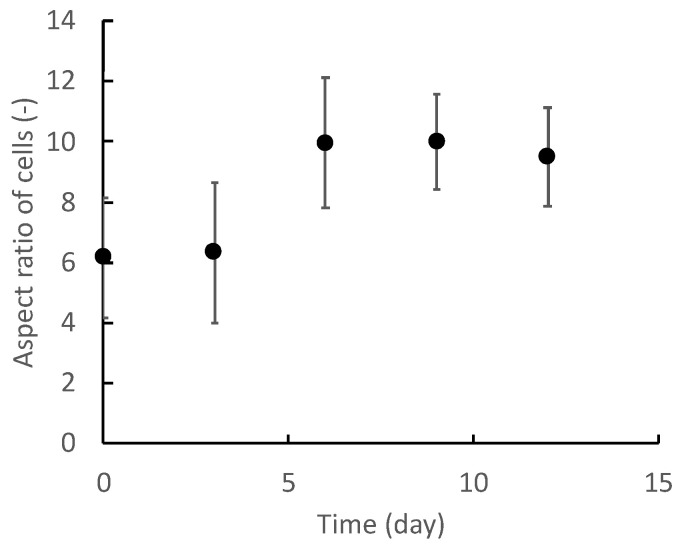
Change in aspect ratio of C2C12 cells during myogenic differentiation culture. Mean +/− SD, n = 25.

Figure 4 shows representative images of differentiated myocytes on each day. The C2C12 cells were elongated by differentiation to form myotube-like morphologies. To label the microscopic images with a differentiation state, cell morphological changes were quantified by the ellipse approximation. Figure 5 and Figure 6 show the cell morphological changes at each point in time during muscle differentiation. The lengths of the major axes of the cells increase from day 0 to day 9, and the rate of increase decreases from day 9 to day 12 (Figure 5). The aspect ratios of the cells increase significantly from day 0 to day 6 and increase slightly from day 9 to day 12 (Figure 6). During the myogenic differentiation of the skeletal muscle-derived myoblast cell line (C2C12 cells), the cells elongate and fuse to form a myotube-like shape. These results suggest that the C2C12 cells undergo myogenic differentiation after 12 days of culturing. Therefore, it is possible to label the microscopic images of undifferentiated and myogenic cells by using the length of the long axis and aspect ratio as indicators.

### 3.2. Influences of Optical Magnification and Cell Morphology in Microscopic Images on Discrimination Accuracy of Cell Discrimination

**Figure 7:**
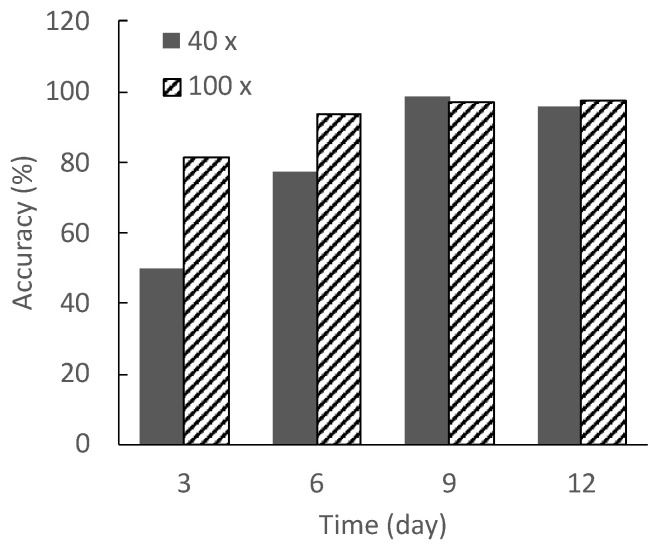
Discrimination accuracy of each microscopic image dataset using our CNN model.

Figure 7 shows the discrimination accuracies of the trained models as created from the datasets of microscopic images at 40× and 100× optical magnifications. The discrimination accuracies of the models created from the datasets at the 40× optical magnification of the microscopic images on days 3 and 6 are 50% and 77.5%, respectively. Notably, the accuracies are greater than 90% when using the dataset of images after day 9. For the training dataset with 100× optical magnification images, the discrimination accuracy of the model generated from the dataset on day 3 is 81.5%, and the accuracy is over 90% after day 6. The discrimination accuracy is lower for both models when using the image datasets from the 40× and 100× optical magnification images on day 3.

As shown in Figure 5 and Figure 6, the differences between the differentiated and undifferentiated cells regarding the long axis length and aspect ratio are small on days 0 and 3. The differences in the morphologies of the cells in the early stage of differentiation and those in the undifferentiated state are also small, as the myogenic differentiation has not yet sufficiently advanced. Therefore, the lower discrimination accuracy on days 0 and 3 is considered to be due to the small differences in cell morphologies. However, on day 6, the discrimination accuracy of the model created from the image dataset at an optical magnification of 40× is approximately 80%, whereas that using the image dataset at an optical magnification of 100× is over 90%. This was due to the difference in the resolution resulting from the optical magnification of the microscope.

**Figure 8:**
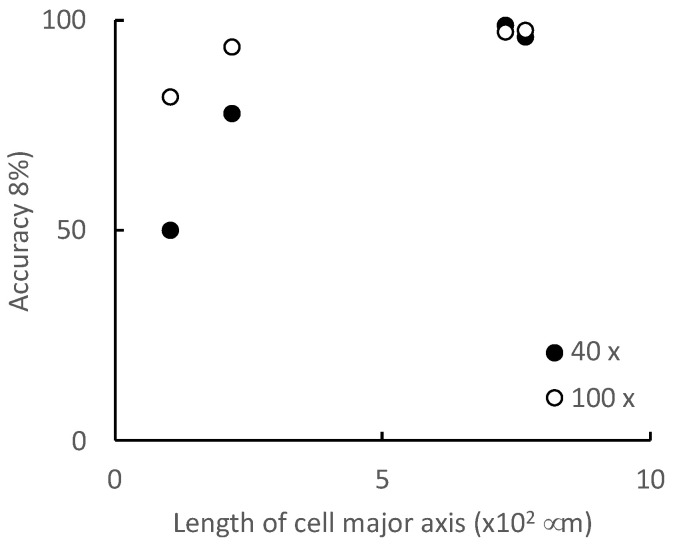
Discrimination accuracy of each microscopic image dataset using our CNN model.

**Figure 9:**
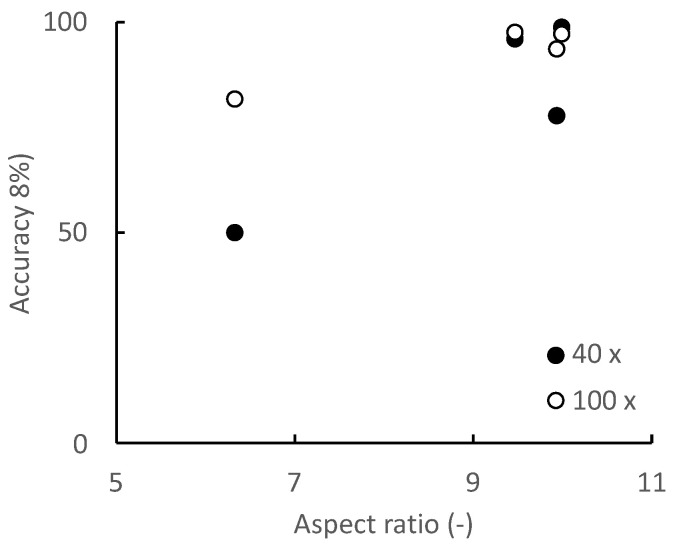
Relationship between the aspect ratio of cells and discrimination accuracy.

To clarify the relationships between the discrimination accuracy and the progression of the morphological changes caused by myogenic differentiation, the relationships between the length of the long axis and aspect ratio of the cell and the discrimination accuracy were evaluated (Figure 8 and Figure 9). For long-axis lengths of up to 300 μm, the discrimination accuracy is lower at the 40× optical magnification than at the 100× optical magnification, indicating that the effect of the optical magnification is significant (Figure 8). As for the aspect ratio, even for image datasets with an aspect ratio greater than 9, which is generally considered to be sufficiently differentiated, the discrimination accuracy is less than 90% when the optical magnification is small (Figure 9). In particular, in the microscopic images on day 6, the aspect ratios of the cells are larger than 9, indicating that the cells have sufficiently differentiated; however, the discrimination accuracy is approximately 80%. This is because the cells on day 6 are small in size with a small long axis length; in this context, the optical image resolution at the 40× optical magnification is insufficient to identify the cells. In addition to the aspect ratio, the long axis length, an indicator of the cell size, has a significant effect on the discrimination accuracy for the myogenic differentiation of C2C12 cells. Furthermore, to increase the discrimination accuracy, the relationship between the image acquiring conditions (e.g., optical resolution, image aspect ratio, etc.) and the CNN architecture should be determined. 

Gao et al. classified microscopic images of human laryngeal carcinoma-derived epithelial cells (Hep-2) into six classes according to their morphologies, and showed a discrimination accuracy of over 90% [13]. As a discrimination accuracy of 90% or higher is generally required for cell image discrimination, it is necessary to acquire microscopic images that enable a discrimination accuracy of over 90% to construct a dataset of microscopic images for training. In this study, to maximize the acquisition efficiency of microscopic images while maintaining a discrimination accuracy of over 90%, it is desirable to acquire microscopic images of cells in which the average length of the long axis is greater than 730 μm owing to myogenic differentiation, using an optical magnification of 40×. However, to identify cells that have undergone myogenic differentiation (even though they have not yet matured to form myotubes) at day 6, it is recommended that one use the 100× optical magnification.

## 4. Conclusions

In this study, the influences of the characteristics of image datasets on the discrimination accuracy of cell myogenic differentiation using a deep learning algorithm were evaluated. The optical resolution of the microscope and cell size had significant effects on the discrimination accuracy of C2C12 myogenic differentiation. Specifically, the discrimination accuracy exceeded 90% even at a low optical magnification (40×) for well-developed myogenic differentiation and advanced myotube-like tissue formation. In the case of cells with advanced myogenic differentiation but without sufficient myotube-like tissue formation, it was necessary to use a relatively high optical magnification (100×) to maintain a discrimination accuracy over 90%.

Finally, when creating a training dataset for cell discrimination using a deep learning algorithm, it is important to adjust the microscopic optical magnification according to the degree of cell differentiation and cell size to improve the time efficiency in image data acquisition.

## References

[1] Konagaya S., Ando T., Yamauchi T., Suemori H., Iwata H. (2015). Long-term maintenance of human induced pluripotent stem cells by automated cell culture system. Sci. Rep..

[2] Maharjan R.S., Singh A.V., Hanif J., Rosenkranz D., Haidar R., Shelar A., Singh S.P., Dey A., Patil R., Zamboni P. (2022). Investigation of the Associations between a Nanomaterial’s Microrheology and Toxicology. ACS Omega.

[3] Diane H.T., Matthew L.W., Joyce Y.W., Margrit B. (2012). Cell morphology classification and clutter mitigation in phase-contrast microscopy images using machine learning. Mach. Vis. App..

[4] Claire L.C., Ata M., Li-chia T., Lan K.B., Allen H., Kayvan R.N., Bahram J. (2016). Deep Learning in Label-free Cell Classification. Sci. Rep..

[5] Boland M.V., Murphy R.F. (2001). A neural network classifier capable of recognizing the patterns of all major subcellular structures in fluorescence microscope images of HeLa cells. Bioinformatics.

[6] Ciresan D.C., Guisti A., Schmidhuber J. (2013). Mitosis Detection in Breast Cancer Histology Images with Deep Neural Networks. Medical Image Computing and Computer Assisted Intervention (MICCAI).

[7] Sadanandan S.K., Ranefall P., Guyader S.L., Wahlby C. (2017). Automated Training of Deep Convolutional Neural Networks for Cell Segmentation. Sci. Rep..

[8] Pedro V., Chris M.B. (2011). A quick, simple and unbiased method to quantify C2C12 myogenic differentiation. Muscle Nerve.

[9] Pennington J., Schoenholz S., Ganguli S. Resurrecting the sigmoid in deep learning through dynamical isometry: Theory and practice. Proceedings of the 31st Conference on Neural Information Processing Systems (NIPS).

[10] Glorot X., Antoine B., Bengio Y. (2011). Deep sparse rectifier neural networks. PMLR.

[11] Oren Z.K., Jimmy L.B., Brendan J.F. (2016). Classifying and segmenting microscopy images with deep multiple instance learning. Bioinformatics.

[12] Krizhevsky A., Sutskever I., Hinton G.E. ImageNet Classification with Deep Convolutional Neural Networks. Proceedings of the 25th International Conference on Neural Information Processing Systems.

[13] Gao Z., Wang L., Zhou L., Zhang J. (2017). Ep-2 Cell Image Classification with Deep Convolutional Neural Networks. IEEE J. Biomed. Health Inform..

